# The role of AI and team topologies in enhancing decision-making flexibility by reducing cognitive overload

**DOI:** 10.3389/fnbeh.2026.1820247

**Published:** 2026-04-13

**Authors:** Hugo Matos-Sousa, Nuno Sousa

**Affiliations:** 1 Coverflex (Universal Cover, S.A.), Braga, Portugal; 2 Centro Universitário de Jaguaríuna (UniFAJ), Jaguaríuna, SP, Brazil; 3 Centro Universitário Max Planck (UniMAX), Indaiatuba, SP, Brazil

**Keywords:** artificial intelligence (AI), behavior, cognitive load, decision-making, team topologies (TT)

## Abstract

Decision-making is a critical skill that impacts the performance of teams, particularly in high-stakes and complex environments, and is known to be affected by cognitive load. When teams face an overload of information and multiple decisions are needed, the process of decision-making becomes slower, performance becomes reduced, and, eventually, there is a burden and exhaustion of the team. This article provides an informed behavioral neuroscience perspective on how psychobiological constraints on behavior, particularly cognitive load, working memory limits, and decision-making under pressure, become relevant in socially organized team settings, and on how the combination of artificial intelligence (AI) with team topologies (TT) may reduce cognitive load and produce an applied framework for understanding the optimization of team architecture and performance by leveraging AI as a tool to reduce cognitive load. By combining a psychobiological perspective with an organizational and translational perspective, the future of the intersection of AI with TT may be generalized and impact teams well-being and performance.

## Introduction

Cognitive load appears within teams whenever the mental (and physical) demands exceed the limits of an individual, or a group of individuals, and leads to impairments of working memory (Sweller, 1988). This situation is quite common in complex contexts that require multitasking and high levels of cooperation between members of teams, and, consequently, performance declines. A common issue for organizations where a large load of work is faced comes not only from its intrinsic complexity but also from the complexity of the systems surrounding the work: unclear decisions, context switching, fragmented information, and dependency management.

In fact, behavioral neuroscience studies have shown that, in conditions of work overload, subjective cognitive load ratings are higher, performance scores are lower, and error rates increase, namely in multitask conditions. In parallel, in complex and demanding situations, and in contrast to less demanding conditions, the activity of the brain, particularly the prefrontal cortex, does not increase, suggesting a “cognitive disengagement” effect, where the brain limits engagement to manage overload (Miller, 1956; Boere et al., 2024). Other studies demonstrated that cognitive load not only affects working memory but also impacts decision-making by increasing decision fatigue and impairing the ability of individuals to take appropriate actions after longer periods of cognitive effort (Stajkovic and Stajkovic, 2025). This condition may lead individuals to become more impulsive and risk-averse and to resort to intuitive, less controlled cognitive processes instead of deliberate ones (Deck and Jahedi, 2015; Zucchelli et al., 2025).

From this lens, the psychobiological foundations of behavior are not confined to the isolated individual, but are also relevant to understanding how humans behave, decide, cooperate, and adapt in socially organized environments, including teams. Thus, team-based work is one of the contexts in which cognitive limitations, decision fatigue, and action-control processes are expressed, amplified, and behaviorally consequential.

This article focuses on how organizations can protect decision-making capacity without slowing delivery. We argue that two levers work best together: first, Team Topologies (TT) reduces coordination overhead by designing team boundaries, responsibilities, and interaction modes around cognitive load as a hard constraint (Skelton and Pais, 2019); and second, AI reduces avoidable mental work by automating repetitive tasks, structuring information, and supporting retrieval and analysis, namely when deployed as part of a socio-technical design rather than a standalone tool. Accordingly, the present article adopts a dual perspective: it is based in behavioral neuroscience, particularly in the psychobiological mechanisms that constrain cognition and behavior under demanding conditions, while also considering the translational relevance of these mechanisms for the organization of team effort through AI and Team Topologies.

### The behavioral neuroscience perspective

Cognitive neuroscience offers important insights into how distinct types of cognitive load influence the behavior of teams. Cognitive load theory (Sweller, 1988) classifies cognitive load into three categories: intrinsic load, extrinsic load, and germane load. Understanding these categories is instrumental in exploring how AI and TT may help teams reduce overload and optimize performance (Table 1). At the same time, these categories are not only useful for describing performance at the individual level, but also for understanding how psychobiological constraints on behavior become expressed in socially organized contexts, namely when individuals must coordinate, decide, and adapt collectively under demanding conditions.

**Table 1:** Summary of the types of mental loads for a team and ways to mitigate them.

Load type	Common drivers	TT levers	AI interventions (examples)	Risks to watch
Intrinsic	Deep domain complexity, tightly coupled decisions; novel problem spaces	Use complicated subsystem teams where justified; time-box collaboration for discovery; clarify ownership	Faster analysis of large datasets; what-if exploration; evidence synthesis	False confidence; hidden assumptions; hard-to-validate outputs
Extrinsic	Dependency thrash; unclear decision rights; meeting overload; interrupt-driven work; poor interfaces	Stream-aligned ownership; platform paved roads; X-as-a-Service interfaces; explicit interaction modes	Triage/routing; alert clustering; thread summarization; action-item extraction	Over-trust in summaries; missing nuance; tool sprawl; new interruptions
Germane	Onboarding; building shared mental models; learning new systems/practices	Enabling teams; facilitating mode; decision logs; stable interfaces to learn against	Retrieval over curated sources; guided onboarding assistants; just-in-time explanations	Skill atrophy; passive learning; stale knowledge bases

The intrinsic component refers to the innate difficulty of a task. Tasks that involve complex decision-making processes, problem-solving, or planning demand significant cognitive resources. It is quite intuitive to imagine that this component amplifies significantly from an individual to a group of individuals. In fact, when teams are asked to manage simultaneously multiple highly complex problems, the intrinsic cognitive load may easily overcome the collective mental abilities and result in cognitive overload. From a behavioral neuroscience perspective, this is particularly relevant because the neural and cognitive systems that support working memory, planning, and flexible decision-making are limited resources, and these limits remain central even when cognition is distributed across a team. Several AI tools have the capacity to reduce intrinsic cognitive load through the automatization of repetitive tasks that increase fatigue and, as a consequence, release the members of the team to higher-rank cognitive tasks such as building hypotheses and planning tasks; an example of this comes from the behavioral neuroscience research field, where the use of a tool named DeepLabCut resulted in significant gains in the performance and analysis of scientific data (Mathis et al., 2018). In this sense, AI may be understood not only as a productivity tool, but also as a factor capable of changing the cognitive conditions under which individual and collective behavior unfold.

The extrinsic (or extraneous) component results from the mental effort that is required to deal with irrelevant or unneeded information that frequently emerges from inefficient systems or from ill-planned workflows. These are very normal when dealing with teams, as it is common to have undefined roles of members of the team, problems with communication within teams, and disorganized workflows. Importantly, from the standpoint of social and organizational behavior, this form of load is highly relevant because it arises not only from the task itself, but from the structure of interaction, coordination, and information flow among individuals. AI can be of help in these contexts by summarizing incidents or long discussions into a timeline, decisions made, and open questions; by extracting action items and owners from operational chatter; by clustering and prioritizing alerts and tickets so humans see the signal first; or by directing requests to the right interface (self-service) or the right team (when escalation is needed). For example, in clinical settings, AI systems can also reduce scanning overhead by prioritizing likely high-risk cases, focusing expert attention where it is most needed (Rajpurkar et al., 2018). The common mechanism is not “better thinking” by the model; it is less time wasted finding the thing worth thinking about. TT addresses extrinsic load at the organizational level by reducing unnecessary dependencies and by making boundaries and interaction paths explicit. Thus, the analysis of behavior becomes relevant not only to how individuals process information, but also to how team environments are organized in ways that either aggravate or reduce unnecessary cognitive burden.

The germane load refers to the cognitive effort dedicated to learning and problem solving. Distinct from intrinsic and extrinsic load, germane load is related to success in the tasks and the innovative process involved. AI can support germane load when it lowers friction in learning, namely through mechanisms that promote retrieval over internal documentation, guided onboarding, and interactive explanations; however, when convenience substitutes for active practice and feedback, understanding may become shallower and skills may deteriorate over time (Kosmyna et al., 2025). The risk is that convenience becomes substitution: if AI answers replace practice and feedback, understanding becomes shallow and skills decay. This point is particularly important from a behavioral neuroscience perspective because it suggests that the use of AI may influence not only efficiency, but also the processes through which individuals and teams consolidate knowledge, develop competence, and maintain adaptive behavioral flexibility over time.

Another point that is relevant to highlight in this neurobiological perspective is the adequate ruling of instrumental behavior. In fact, the critical challenge in instrumental behavior relies on the balance between goal-directed behaviors and habits; this holds true both at the individual and at the team level. The activation of distinct corticostriatal networks in ruling such behaviors has been well established. While habits are typically viewed as less cognitively demanding, goal-directed behaviors imply greater focus and cognitive effort, including the activation of the prefrontal cortex (Graybiel, 2008; Dias-Ferreira et al., 2009). Importantly, the adequate transfer from a goal-directed action to a habit and vice versa is critical for proper decision-making and underlies the flexibility that is needed to adjust behavior to ever-changing circumstances. Several factors, such as risk aversion, impulsivity, and stress, are known to modulate such dynamics in the activity of corticostriatal circuits and in the processes of decision-making. In team settings, these dynamics may also acquire a social and organizational dimension, since overload, poor coordination, and excessive uncertainty can favor less flexible and more automatic forms of responding, whereas well-structured environments may help preserve goal-directed control and adaptive decision-making. Our argument is that AI has the possibility to also interfere in these dynamics by augmenting the appropriate use of habitual networks, which require less cognitive effort, and, by doing so, to significantly diminish cognitive load. Accordingly, the relevance of AI in this context is not limited to task acceleration, but extends to the possibility of modulating the balance between effortful and less effortful modes of behavioral control in ways that may be beneficial or, if poorly implemented, potentially maladaptive, for both individuals and teams.

### Team topologies: a framework to optimize the structure of teams

Team Topologies (TT) treats cognitive load as a design constraint: teams should be sized and shaped so they can understand, operate, and improve their part of the system without drowning in coordination (Skelton and Pais, 2019). TT provides four team types and a set of interaction modes (notably collaboration, facilitating, and X-as-a-Service) to control how work and knowledge flow across boundaries.

The stream-aligned teams are autonomous sub-teams designed to focus on a specific value, allowing other teams to work independently while still interacting with each other. Such autonomy helps teams in decision-making and in the execution of tasks, without the need for excessive coordination, and promotes focus on their main goals and objectives. A simple example is a team that owns user onboarding from start to finish. They handle everything from sign-up and identity checks to the moment a user first gets real value. That includes product changes, tracking and analytics, experiments, and keeping the flow healthy in production. Because one team is responsible for the whole journey, most decisions can be made where the work happens. There is less back-and-forth between departments, fewer handoffs, and fewer meetings just to stay aligned. As a result, the team moves faster and spends less energy coordinating.

Enabling teams offer support and specialized expertise to stream-aligned teams, reducing obstacles and enhancing performance. These teams offer support on demand, avoiding overload of information in stream-aligned teams. In addition, by providing guidance and expert skills, these teams create a proper balance of cognitive load within the organization. Obviously, AI tools and approaches may add value to these processes by providing tools that expand expert knowledge and solutions to the stream-aligned teams.

The complicated subsystem teams manage specialized subsystems or technical components. Even though these teams provide specialization, their interactions with other teams may produce cognitive load if not properly supervised. This is where AI tools may be of significant help because they may determine the proper workflow of such information to other teams.

Finally, the platform team builds and manages the platforms that support the work produced by stream-aligned teams. By providing the platforms and the tools, they regulate the access of stream-aligned teams to the resources without engaging in complex tasks. AI features can be part of the platform (e.g., incident summarization, anomaly detection, or ticket triage), but the platform team’s role remains the same: build and run a dependable product that stream-aligned teams can use without interrupts, surprises, or hidden operational work.

### Integration of AI and behavioral neuroscience in favor of teams’ performance

In this section, we move from literature review to conceptual synthesis. Because direct empirical research specifically examining the integration of AI with Team Topologies is still limited, we outline a research-informed framework and identify practical hypotheses and priorities for future study.

The incorporation of AI tools into the workflow of teams can be instrumental in reducing their cognitive load (Dunis et al., 2017). By automatizing routine tasks, by offering support to decision-making processes, and by facilitating communication between teams, AI enables members of teams to focus on higher cognitive tasks that require strategic reasoning and outcome-oriented behaviors. AI systems could crucially support decision-making processes through the analysis of big data in real time that allows teams to take more evidence-based decisions, to anticipate and mitigate unnecessary risks, and to suggest better and broader solutions in distinct contexts.

Emerging evidence suggests that AI is most useful when it is integrated into workflows and socio-technical systems, rather than deployed as an isolated add-on tool (Salwei and Carayon, 2022; Gates et al., 2026); however, the strength of evidence varies by domain, and poorly designed workflows may simply shift rather than reduce cognitive burden (Shalu et al., 2026). If the workflow is already messy, AI often just moves the burden around. People switch context more, spend time verifying outputs, and take up new rules and processes. Finally, but of the utmost importance, the AI-powered tools to optimize communication should be highlighted, as they represent a plus in teams’ interaction and in keeping the track record of the processes, a task that ultimately consumes a significant amount of energy and cognitive effort.

Of course, AI adoption is not without risks. The dangers it introduces are real and could impact the teams’ capabilities. Automation bias is the obvious one: when outputs sound confident, people trust them more than they should and stop checking edge cases (Parasuraman and Manzey, 2010). Another is verification overhead: if every AI suggestion needs a careful review, the time saved upfront can come back later as extra cognitive work, especially when correctness is hard to validate quickly. Finally, there is tool sprawl and governance drag, including new systems to operate, permissions to manage, security reviews, logging, evaluation, and the inevitable questions about accountability when something goes wrong.

The practical takeaway is to treat AI like any other dependency in your system. Keep its scope tight. Be explicit about where it is trusted and where it is not. Make failure modes visible. Measure quality. And have a clear escalation path when outputs conflict with observed reality or when the stakes are high. TT makes this easier because it gives a place to “put” the capability. A common pattern is for the platform team to ship AI as a service: approved models, shared prompt templates, retrieval over trusted sources, observability, auditability, and safe defaults. The goal is not to turn the platform team into an AI helpdesk; it is to make the capability self-service, reliable, and governed in one place. Stream-aligned teams can then use those services inside their own workflows, things like incident response, support triage, and release readiness, without giving up accountability for outcomes. They decide when to rely on the tool, when to ignore it, and when a situation belongs in the “slow thinking” lane. Enabling teams play a different role: they help other teams adopt AI responsibly. That often means coaching teams on what “good use” looks like, where models tend to mislead, how to evaluate outputs, and how to avoid swapping real learning for convenience. They can also do more than coaching. Enabling teams are well placed to prepare assistant AIs to use approved sources of information, so teams spend less time digging for basics and repeating the same onboarding explanations. Finally, complicated subsystem teams matter whenever the domain has sharp boundaries. Their job is to protect interface integrity, so AI-assisted workflows do not blur what is true, what is assumed, and what is merely plausible. Framed this way, AI stops being an individual productivity trick and becomes an organizational capability. It has clear ownership, clear interfaces, and clear constraints, which is usually the difference between a tool that quietly helps and a tool that quietly drains attention.

## Conclusion

The integration of AI and TT, based on the evidence provided by behavioral neurosciences, may guide the future of the workflow for individuals and, most importantly, for teams. We have argued that the combined use of these approaches permits leveraging the use of AI automatisms and the increased focus of team members on goal-directed actions while managing the risks of AI adoption. When combined with proper team structure through TT, such efficacy augments may result in performance optimization and ultimately in shaping teams’ behavior and dynamics in progressively more demanding and complex ecosystems.
